# Developing a Tool for Increasing the Awareness about Gendered and Intersectional Processes in the Clinical Assessment of Patients – A Study of Pain Rehabilitation

**DOI:** 10.1371/journal.pone.0152735

**Published:** 2016-04-07

**Authors:** Anne Hammarström, Maria Wiklund, Britt-Marie Stålnacke, Arja Lehti, Inger Haukenes, Anncristine Fjellman-Wiklund

**Affiliations:** 1 Department of Public Health and Clinical Medicine, Unit of Social Medicine, Umeå University, Umeå, Sweden; 2 Umeå Centre for Gender Studies in Medicine, Umeå University, Umeå, Sweden; 3 Department of Community Medicine and Rehabilitation, Physiotherapy, Umeå University, Umeå, Sweden; 4 Department of Community Medicine and Rehabilitation, Rehabilitation Medicine, Umeå University, Umeå, Sweden; 5 Department of Clinical Sciences, Unit of Professional Development, Umeå University, Umeå, Sweden; 6 Department of Public Mental Health, Division of Mental Health, Norwegian Institute of Public Health, Kalfarveien 31, Bergen, Norway; 7 Research Unit for General Practice, Uni Research Health, Kalfarveien 31, Bergen, Norway; Iran University of Medical Sciences, ISLAMIC REPUBLIC OF IRAN

## Abstract

**Objective:**

There is a need for tools addressing gender inequality in the everyday clinical work in health care. The aim of our paper was to develop a tool for increasing the awareness of gendered and intersectional processes in clinical assessment of patients, based on a study of pain rehabilitation.

**Methods:**

In the overarching project named “Equal care in rehabilitation” we used multiple methods (both quantitative and qualitative) in five sub studies. With a novel approach we used Grounded Theory in order to synthesize the results from our sub studies, in order to develop the gender equality tool. The gender equality tool described and developed in this article is thus based on results from sub studies about the processes of assessment and selection of patients in pain rehabilitation. Inspired by some questions in earlier tools, we posed open ended questions and inductively searched for findings and concepts relating to gendered and social selection processes in pain rehabilitation, in each of our sub studies. Through this process, the actual gender equality tool was developed as 15 questions about the process of assessing and selecting patients to pain rehabilitation. As a more comprehensive way of understanding the tool, we performed a final step of the GT analyses. Here we synthesized the results of the tool into a comprehensive model with two dimensions in relation to several possible discrimination axes.

**Results:**

The process of assessing and selecting patients was visualized as a funnel, a top down process governed by gendered attitudes, rules and structures. We found that the clinicians judged inner and outer characteristics and status of patients in a gendered and intersectional way in the process of clinical decision-making which thus can be regarded as (potentially) biased with regard to gender, socio-economic status, ethnicity and age.

**Implications:**

The clinical implications of our tool are that the tool can be included in the systematic routine of clinical assessment of patients for both awareness raising and as a base for avoiding gender bias in clinical decision-making. The tool could also be used in team education for health professionals as an instrument for critical reflection on gender bias.

**Conclusions:**

Thus, tools for clinical assessment can be developed from empirical studies in various clinical settings. However, such a micro-level approach must be understood from a broader societal perspective including gender relations on both the macro- and the meso-level.

## Introduction

Women’s subordinated position in society including in health care has received increasing attention around the world and has highlighted the need to emphasize gender equity and gender equality in health care [1, 2]. As a response gender policies have been developed in order to tackle these problems. In e.g. Canada, the government uses a sex and gender-based analysis to develop, implement and evaluate research, programs and policies in order to address the different needs of women and men [3]. WHO has developed a Gender Policy ‘to ensure that all research, policies and programmes/projects in WHO are designed from a gender perspective, and that this is accomplished in a systematic and sustainable manner [4].

The terms gender equality and gender equity have different meanings but are sometimes used interchangeable. In this paper we use gender equality to define the absence of discrimination between women and men [5]. On the other hand we define gender equity as focusing on meeting women’s and men’s health needs, whether similar or different which is not the main interest in this paper. Intersectional approaches have emphasized the need to recognize the heterogeneity within groups of ‘men’ and ‘women’ which means that men and women differ with regard to multiple, intersecting dimensions, such as class/education, ethnicity and age [6]. Gendered analyzes need to go beyond additive analyzes of the combinations of gender, class, age etc. and instead focus on the complex intersections between the major factors which potentially influence gendered health [5].

There are a large number of studies indicating that there is gender bias, i.e. an unintended and systematic neglect of one gender, in health care [7–13]. Women are more often neglected than men and medically unjustified differences in the availability of examination and treatment of women have been demonstrated in connection with a number of different diseases, such as coronary heart disease [7, 13–15], neck pain [16], irritable bowel syndrome [17], tuberculosis [18] renal transplantation as well as HIV treatment [7]. Most of the publications on gender bias use a dichotomous view on men versus women rather than an intersectional approach.

There is a lack of scientific publications about strategies to combat gender and social bias in health care. Many tools have been developed in order to analyze gender equality in health policies on the macro-level [19]. The tools have been developed to support problem identification through a series of questions—that empirical data can provide answers to—which help detect processes of gender inequality [19, 20]. Thus, the tools can provide an increased understanding of a problem or give examples of activities that could be undertaken to address gender inequality. These tools often follow certain interrelated stages of policy analysis, like the following: 1) explore the issue and how it is represented; 2) define goals and objectives; 3) research and consultation; 4) develop and analyze options; 5) make recommendations; 6) communicate the policy [21, 22].

A WHO report has reviewed 17 widely used gender tools and their usefulness in gender analyzes in health [19]. The report concludes that the tools do not provide methodologies, details of how pilot interventions could be extended or other practical ‘how to’ guidelines for taking action. The analyzed tools raise questions about quality of care but the questions are more focused at a macro- than at the micro-level of actual health service delivery.

Thus, there is a need for developing tools which address gender and social inequality in the everyday clinical work in health care. One clinical study in the field is the so-called “laundry bag project” which discovered gendered standards for dermatological treatment of common diagnoses. The study showed that among patients referred to a dermatological clinic with diagnoses of psoriasis or eczema [23] men received more help with emollients than did women. One of the conclusions from the project was that clinical tools to evaluate disease severity during the treatment could reveal possible gender bias. The WHO report also concludes that identifying gender issues in health care services requires a detailed in-depth investigation [19].

Our paper takes its point of departure in such detailed investigation in own sub studies which synthesize the results of our overarching research-project “Equal care in pain rehabilitation” about gendered and social processes in the case of pain rehabilitation. The aim of our paper was to develop a tool for increasing the awareness of gendered and intersectional processes in clinical assessment of patients, based on the case of pain rehabilitation.

The tool is aimed at professionals of pain rehabilitation in both primary and specialist care for avoidance of gender bias in their assessment and selection of which patients with chronic pain that should be referred to multimodal rehabilitation programmes.

## Methods

### The overarching research-project “Equal care in pain rehabilitation”

We designed a research project “Equal care in pain rehabilitation” aiming at developing a tool for critically scrutinizing and increasing awareness about gender equality, which could be used by professionals in daily health care. In the overarching project we used multiple methods [24] including quantitative and qualitative methods, in five sub studies, in order to develop the gender equality tool. The gender equality tool described and developed in this paper is thus based on results from our sub studies about the processes of assessment and selection of patients in pain rehabilitation [25–29]. The population and method of these earlier sub studies are described below.

### Ethical statement

Written and signed informed consent was obtained from all participants of the sub studies. The whole project was approved by the Regional Ethics Vetting Board in Umeå, Sweden.

### Setting

The study was conducted at the Pain Rehabilitation Clinic at Umeå University Hospital as well as at a Primary Health Center in Umeå, Sweden. Patients with chronic pain were referred from primary health care to the Pain Rehabilitation Clinic. Individual plans for rehabilitation were based on two days assessment of the patients by interdisciplinary teams (consisting of specialist physician in rehabilitation medicine, physiotherapists, social workers, occupational therapists and psychologists) and information about the patient from self-administered questionnaires. Individuals who were recommended multidisciplinary rehabilitation were offered enrolment in a 4-week inpatient multidisciplinary pain rehabilitation programme at the Pain Rehabilitation Clinic. The program focused on pain management and education about pain and its consequences. Almost everyone, who was selected to the rehabilitation programs, participated [30]. Patients who were not referred to these programs were followed up in primary health care with further rehabilitation intervention by a single profession.

Inclusion criteria for referral to the multidisciplinary rehabilitation programme were (*i*) disabling, non-malignant, chronic and complex musculoskeletal pain (on sick leave or experiencing major interference in daily life due to chronic pain); (*ii*) age 18–65 years; *(iii)* no further medical investigations needed; *(iv)* written consent to participate in and attend the multidisciplinary programme; *(v)* agreement not to have parallel contacts with therapists such as physiotherapists while attending the multidisciplinary pain rehabilitation programme.

### Design and data collection in earlier sub studies

#### Population

The population for the quantitative analyzes consisted of consecutive patients (n = 595 women, 266 men) referred mainly from primary health care centers to the Pain Rehabilitation Clinic and assessed between 5 November 2007 and 13 December 2010.

The population of the qualitative analyzes consisted of patients referred to the Pain Rehabilitation Clinic. A strategic selection (n = 10) was made in relation to gender, age and country of birth. A purposeful sampling was also performed, consisting of health care professionals at the Pain Rehabilitation Clinic (n = 7) as well as general practitioners and their trainees working at one primary health care center in Umeå (n = 8).

#### Analyzes of our earlier quantitative and qualitative sub studies

For the quantitative analyzes, patient data were collected from the Swedish Quality Registry for Pain Rehabilitation [30] and linked to the patients’ individual records containing the final decision on being selected or not to multidisciplinary rehabilitation programs. The register is mainly based on patients’ information from validated self-administered questionnaires completed before the first multidisciplinary assessment [30]. Multiple logistic regression analyzes were used to examine the association between being low educated women [25] respectively pain severity [27] for selection to pain rehabilitation and gender [26] for further treatment in primary care among those not referred to pain rehabilitation.

For the qualitative analyzes, individual semi-structured interviews were performed with patients as well as with health care professionals at the Pain Rehabilitation Clinic. A focus-group discussion was performed with general practitioners with focus on experiences of possible in/equalities in health care.

The co-authors worked together during the whole project period with various responsibilities for the sub studies. For this paper all authors contributed from their various disciplinary, theoretical and methodological backgrounds in a step-wise process throughout the time of the project.

### Developing the gender equality tool

To develop our gender equality tool we took inspiration from earlier tools, especially the following questions from the Victorian lens [20]:

How will women and men from diverse backgrounds be meaningfully consulted about the issue?

Are there particular cultural protocols to consider?

Are under-represented groups being considered?

What are the ‘non-negotiables’ for consultation?

How will expectations and conflicting interests be managed?

These questions were further developed inductively with Grounded Theory (GT) [31] into the area of clinical decision-making in pain rehabilitation based on the findings of our five sub studies [25–29]. The GT approach is suitable to mirror concrete observations from the field, such as pain rehabilitation, and to synthesize and conceptualize the observations on a more abstract theoretical level [32, 33]. Each of the questions above was used to search for findings and concepts [32, 33] related to gendered and social processes in pain rehabilitation, in each of our sub studies. For example, the question *Are under-represented groups being considered*? was posed and we found the importance of education in one quantitative [25] and in one qualitative sub study [28]. This led us to develop several questions about socioeconomic status.

Through this process, the actual gender equality tool, consisting of questions about the process of gendered and social clinical assessment, was developed (see Table 1).

**Table 1:** A tool for analyzing gendered and social processes in the clinical assessment of pain patients. The follow-up question “If yes, why?” is relevant in most questions.

Questions to the clinic/health care centre	Is it possible to access rehabilitation programs, if you do not speak Swedish [28]?
	Is information about the programs available in other languages than Swedish [28]?
	Should pain intensity be a criterion for rehabilitation [27]?
Questions to the professionals	Are we likely to choose patients who are similar to us in the rehabilitation team? [28]
	Are we influenced by patients’ socioeconomic status, by their verbal skills and by their dress and appearance? If yes, does this apply to women as well as men, and higher educated as well as lower educated [28, 29]
	Do we refer low-educated women less often to rehabilitation than high-educated women? [25]
	Do we consider that low-educated women have lower possibilities than others to benefit from the rehabilitation programme? [25]
	Do we have (unspoken) demands on cognitive ability to make use of the rehabilitation? [25]
	Do we treat men and women differently in spite of the same pain problems? For example, are men more often than women referred to physiotherapist? [26]
	Do we believe that men have higher demands on investigation and referrals than women? [26]
	Do we investigate men more in depth than women, independently of pain severity? [26]
	Do we take pain amongst men more seriously than among women? [26]
	Do we consider pain among men as more severe than among women? [27]
	Do we believe that men are more resistant to pain than women? [27]
	Do we consider it as an advantage to be work oriented (i.e. unemployed, house wives and those who receive social security benefits are less likely to be prioritized to rehabilitation) [28]?
	Do we believe that it is an advantage to be able and willing to behavioural change? If yes, does this apply to women as well as men, and higher educated as well as lower educated [28]?

### Developing a comprehensive model

As a more comprehensive way of understanding the tool, we performed a final step of the GT analyses. Here we synthesized the results of the tool into a model.

The model illustrates a process of assessment and selection of patients with chronic pain as a funnel, a top down process governed by gendered and intersectional attitudes, rules and structures as well as clinical decisions.

To summarize, GT is a flexible and emergent research design [33] which in our study is illustrated by the step-wise analytical process resulting in a tool and finally in a model. GT is often used to capture social processes or phenomena in line with our focus on potential discrimination and inequalities in a rehabilitation context [33].

## Results

### Summary of the findings of our sub studies

Below the findings from our quantitative and qualitative sub studies are summarized. Low-educated women were less often selected to multidisciplinary rehabilitation programs than high-educated women (OR 0.55, CI 0.30–0.98), even after control for age, being born outside Sweden, pain intensity and a measure of anxiety and depression [25]. No significant findings were found when comparing the results between high- and low-educated men.

Men were more often than women referred to physiotherapy and x-ray, independent of self-reported pain intensity, pain activity and pain localization [26].

Higher scores on self-reported pain did not guide selection to multimodal rehabilitation [27]. In fact, a negative trend was found among women. The higher the scores of pain, the less likely were women to be referred to multimodal rehabilitation.

The interviews with the GPs resulted in one core category “Via Dolorosa” which captured the social process over time through which potential inequalities were created along patients’ passage from primary care to assessments in specialist rehabilitation clinic [28]. The core category included the four subcategories “Pain—an illness with low status”, “A proper patient on the go”, “Stereotyping thoughts” and “Assessing and selecting in context”.

The analysis of patients’ narrated experiences from assessment and treatment at a multidisciplinary pain rehabilitation clinic, including perceptions about inequalities, resulted in one main theme “Accessing rehab–not a given” and three interrelated categories: “The power of diagnosis”, “The power of gender”, “The power of social status” [29]. The results show potential inequalities related to patients’ access to rehabilitation resources.

### The gender equality tool

Table 1 shows the gender equality tool, which was inductively developed from our sub studies. The tool poses three questions to the clinic/health care centre followed by 12 questions to professionals. Most of the questions can benefit from further reflection about the possible reasons behind the answers. Therefore, a follow-up question “If yes, why?” is relevant in most of the questions.

### A comprehensive model

**Fig 1:**
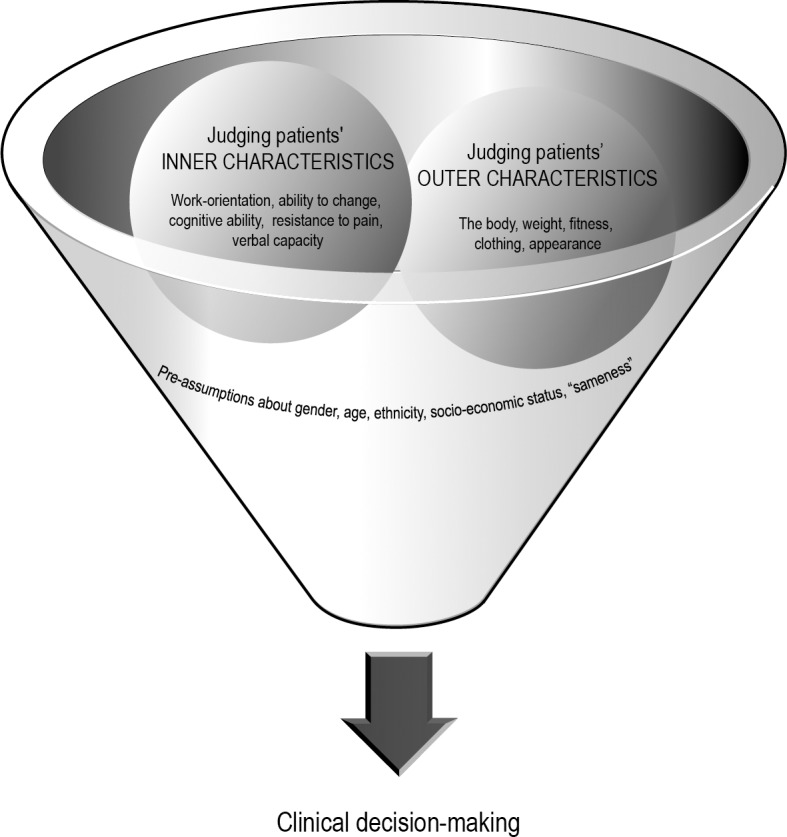
A comprehensive model for illuminating the top down process of clinical decision-making performed within a context permeated by intersectional and gendered attitudes, rules and structures.

To further understand the tool we synthesized our empirical findings on a more abstract level. In this way a comprehensive model was developed as a final step in our GT analyses (Fig 1). The model illuminates the clinical process of assessing patients with chronic pain. The process is illustrated by a funnel with a top down process of clinical decision-making performed within a context permeated by intersectional and gendered attitudes, rules and structures. The model includes two dimensions, *judging patients’ inner characteristics* and *judging patients’ outer characteristics*, which are related to several possible discrimination axes such as gender, socio- economic status, ethnicity and age.

Professionals were interpreted as prone to select patients for further rehabilitation who were similar to themselves, for instance in terms of verbal skills or cognitive ability [28]. The selection process was thus informed by social processes which can be referred to pre-assumptions of “sameness” and “otherness” [28]. Choosing similarity means that the staff would rather choose someone similar to themselves (in relation to not only sex/gender but also education and ethnicity) for further rehabilitation. In spite of having similar needs, patients who are white, educated, well-dressed, Swedish speaking men may be judged as being more work-oriented, flexible and having cognitive abilities which would favor them in the rehabilitation process. These notions were confirmed by patients’ perceptions [29], as well as by the quantitative results [25].

Thus, our model can be used to show how clinical assessment and the selection process of patients to pain rehabilitation, constitute a (potentially) biased and gendered process of judgment of patients’ inner and outer characteristics in relation to (potential) discrimination axis.

## Discussion

Our paper takes a novel approach by using own empirical data and GT analyzes about gendered and intersectional processes in pain rehabilitation in order to develop a tool for increasing the awareness of gender equality in the assessment of patients with chronic pain. Our approach is also novel as we include intersectional dimensions in our analyses of gender equality. The tool is developed as an illustration of important gendered and intersectional processes in the clinical assessment of patients with chronic pain. The model gives a more comprehensive understanding of the tool. Our study could be seen as a study of pain rehabilitation but the gender equality tool may also be useful in assessment and selection of patients with other diagnoses. We welcome research which tests our tool in assessment of patients with other diagnoses as well as in other settings. Our tool could be used for both awareness raising and for avoiding gender and social bias in clinical decision-making.

The WHO report on tools for developing gender equality in health concludes that further work is needed about useful methodologies on ‘how to’ go about addressing gendered health issues [19]. Our tool is such a contribution, illustrating how clinicians can decrease the risk of gender inequality in the clinical decision-making process by being aware of gendered processes. Overall, our paper is a contribution to the field as there is a lack of tools for analyzing gender equality in clinical practice in health care.

Our tool is one of the first to provide guidelines for the micro-level, i.e. for clinical work. The Victorian lens [20], which inspired our analyses, has developed clinical relevant questions (tool) in one (Research and consultation) out of five steps in their check-list. Other tools, such as The BIAS FREE Framework [34] is about eliminating gender bias in health research and not relevant for clinical work. The WHO report [19] concludes, after reviewing 17 widely used gender tools in health from around the world, that the tools are helpful in identifying gender issues in health that need to be addressed but the tools do not provide methodologies or other practical ‘how to’ guidelines for taking action in clinical work. The micro-level approach for practical clinical work is the contribution of our tool. Our paper is situated on the micro-level (patients and clinicians) and a contribution to the field is that we explore the patient’s point of view. According to Annandale and Kuhlman we need a better understanding of the experiences of the patients’ views and experiences of the various ways in which gender permeates their meetings with the healthcare [35]. Annandale and Kuhlman also call for more context-sensitive empirical studies [35].

But our findings must be understood from a broader societal perspective including also the meso- and macro-levels. All societies have a gender order, constructed by multiple ideas about what is seen as feminine or masculine, in which male domination is created and maintained [36]. The treatment of patients may be gendered due to the fact that clinicians (as everyone else) are influenced by men’s higher status in society and the unequal gendered power relations in society. The gender order on the macro-level in society [37] is built up by the relations between men and women in production (where e.g. the gender order is visualized as the extremely gender-segregated workplace in health care), in emotional relations (e.g. ill-health related to emotional domestic work is less recognized and attended to than ill-health related to waged work) as well as to symbolic relations (in which qualities, attributes and behaviors are assigned to men and women respectively).

Gender relations on a macro-level are of importance for all other gender relations in society [37]. On this level, there are many policies about the importance of gender equality in health care around the world [4, 38] and as Annandale and Kuhlman state ([35] p 460): “The discussion of the macro-context has highlighted the array of factors that need to be taken into account when considering the obstacles to gender appropriate health care.”

On the meso-level, i.e. the organizational level of the health care system, there has been much discussion about and development of gender mainstreaming (such as [39–41]). However, there is a lack of knowledge about the importance of attitudes towards gender mainstreaming among top managers in in health care, and the importance of these attitudes for successful implementation of mainstreaming [32].

Our tool could also be discussed within a gender theoretical framework. Our social constructivist perspective on gender and health is based on the belief that we live in historical, social and cultural contexts that influence the way we write, think and talk about gender and health [42, 43]. Thus, we regard gender as an ongoing activity embedded in everyday social interactions, shown in our ways of acting, talking and narrating about life. In our tool, this is illustrated as gendered processes in everyday clinical decision-making. As individuals, we try to imitate society’s ideal picture of how to be a man or a woman; but, due to the different classes and subcultures that we live in, individuals succeed in this to a greater or lesser extent [42, 43]. Also, as clinicians we may tend to prioritize patients who behave like an expected man or woman. Research into constructions of masculinities and femininities has emphasized their internal complexity, capacity for change and interweaving with other social relations, for example of class and ethnicity [44].

For example, the laundry bag project shows how personnel within healthcare continuously reconstruct what is seen as masculine or feminine in society through treating men and women differently [23]. This is in line with a study on gender constructions in health care [45], which shows how staff constructed male patients as stubborn and unwilling to seek help whilst constructing female patients as over-users of health care services. The health professionals criticized culturally idealized forms of masculinity for their role in men’s reluctance to seek help, and constructed women’s problems as more trivial than men’s [45]. Connell’s theory of hegemonic masculinity [44] has been used to critically examine how men’s underutilization of medical services may be influenced by the social construction of masculine identities [46]. By using biomedical and morality discourses the men maintained a masculine identity when narrating about their health-seeking behavior in order to identify themselves as legitimate users, as they position women as frequent and trivial users of health care [46]. These results support the utility of hegemonic masculinity as a theoretical basis for examining the construction and maintenance of gendered identities by highlighting the complexity of multiple masculine identities. Multiple dimensions of femininity may also be relevant to the construction of feminine identities in relation of women’s experience of care, although this has not been researched to date.

Our results can be seen as constructions of various masculinities and femininities in process of clinical assessment of patients in pain rehabilitation. In this process, gendered attitudes and judgements take place. Our gender theoretical approach goes beyond a dichotomous view on gender as we bring in other power dimensions, such as social class and ethnicity in their interaction with gender. The various axes of power do not necessarily act in unison—but operate in complex ways–upon health. One category, such as ‘class’, takes its meaning from another, such as ‘gender’, and a ‘new uniquely hybrid creation’ emerges at the intersection which becomes the unit of analysis [47].

### On the methods

The strength of our paper is the width and depth of our study–both in relation to data (both patients and professionals), methods (qualitative, quantitative) and contents (the whole chain of rehabilitation). Our approach is a novel way of synthesizing the findings from empirical studies in the field into a clinically relevant tool. Triangulation between researchers, from perspectives of physiotherapy (AFW, IH, MW), rehabilitation medicine (BMS), and general practice (AH, AL) was used to increase the credibility in the data analysis [33]. The emerging result was discussed repeatedly in the research group, during the development of the tool and the model for final negotiated outcomes.

A methodological challenge was the lack of established methods for synthesis of earlier findings. With only quantitative papers we could have performed a meta-analysis and with only qualitative papers we could have done a meta-synthesis. However, we wanted to use all our data and therefore chose GT to support the step-wise analytical process of synthesizing and abstracting empirical findings from different sources. GT also proved to be suitable as it has the potential to capture and theorize social processes [33].

In the overarching project we used multiple methods as we had both quantitative and qualitative approaches attached to an overall inductively derived aim [48]. In contrast to a mixed-methods design the research questions for each sub study were separate, but complementary to our aim [48]. A mixed-methods design could explore multidimensional understanding of gender equality in the assessment process which is interesting but not the aim of this study. The innovative contribution of our paper is the gender equality tool, which needs to be tested in other studies of clinical assessment of patients with chronic pain. Future research is also needed about tools in other clinical settings as well as in other cultural contexts.

The empirical data of our sub studies were collected from one region in Sweden. During the three-year inclusion period of patients for the quantitative studies, the procedures for multidisciplinary team assessments did not change, thus enhancing the reliability of the data. In addition the team assessment was performed by experienced professionals with high staff continuity during the data collection period. Further, patient data were collected from a national register for pain rehabilitation which includes approximately 80% of pain management programs in Sweden [30]. The procedure used by the multidisciplinary team for selection of patients for multidisciplinary rehabilitation is similar throughout Sweden; thus, we can assume that the generalisability of the study is good on a national level. Moreover, since comparable multidisciplinary assessment and selection visits often precede participation in rehabilitation programs in other counties as well [49], and since the questionnaires of the register have been widely used for measuring chronic pain, depression and anxiety in a range of pain rehabilitation contexts [30, 50], we can assume that the generalisability of the quantitative sub studies is good to countries with similar organization for the rehabilitation of patients with chronic pain. The findings of the qualitative sub studies can be transferrable to similar settings.

The prevalence of pain is systematically higher among women than in men [51] and as a consequence more women than men are referred to rehabilitation. This may have influenced the findings in one of our quantitative sub studies [25]. As we discuss in that study the lack of significant findings between educational level and selection for multidisciplinary rehabilitation among men may be due to a type 2-error. Thus, we cannot conclude that there are no educational differences in pain assessment among men.

Despite a relatively small sample of interviews, we believe that the results of our qualitative study about staff experiences reflect crucial aspects of perceived inequalities, which can be further deepened and discussed in relation to other rehabilitation contexts in future studies.

### Clinical implications

The main clinical implication of our study is that our tool can be included in the systematic routine work of clinical assessment of patients as well as in decisions about criteria for inclusion and exclusion to rehabilitation programmes. The tool questions to the workplace could be asked in the systematic evaluation of the work environment, which for example according to Swedish law must be performed regularly at work places. These evaluations include surveys of the work place activities, such as consultations of patients, with the aim of providing gender equal care. The tool questions to professionals could be asked for each patient by both those who make the clinical assessment and those who treat the patients. The tool could also be used in team education for health professionals as an instrument for reflection of gender bias.

## Conclusions

Gender equality tools for clinical assessment can be developed from empirical studies in various clinical settings. Such a micro-level approach must be understood from a broader societal perspective including gender relations on both the macro- and the meso-level.
